# Infection, Healing, and Cost in Open Fractures: A Review of Negative Pressure Wound Therapy

**DOI:** 10.7759/cureus.93045

**Published:** 2025-09-23

**Authors:** Chan Khin, Olive Kyaw

**Affiliations:** 1 Trauma and Orthopaedics, University Hospitals Sussex NHS Foundation Trust, Brighton, GBR

**Keywords:** bone healing, cost-benefit analysis, fracture fixation, negative-pressure wound therapy, open fracture, orthopaedics procedures, soft tissue injuries, surgical wound infection, vacuum-assisted closure (vac)

## Abstract

Complex open fractures present substantial challenges, including infectious complications, healing delays, and functional impairment. Sub-atmospheric pressure therapy has gained adoption as a supplementary treatment modality, despite ongoing debate regarding its clinical efficacy.

A focused literature search was conducted in PubMed, Google Scholar, and the Cochrane Library (1997-2025). Randomised controlled trials, cohort studies, and systematic reviews on negative pressure wound therapy (NPWT) in open fractures were prioritised. Outcomes of interest included infection rate, healing time, flap/graft requirement, reoperation rate, and cost-effectiveness.

Early randomised and cohort studies demonstrated reduced deep infection rates with NPWT compared with standard dressings. Pooled analyses demonstrated reduced infection and flap failure rates, although union times and reoperation rates showed no consistent benefit. The WOLLF multicentre trial found no improvement in long-term functional outcomes, and an accompanying economic analysis questioned cost-effectiveness.

NPWT appears most useful in severe open fractures with significant soft-tissue loss, where it may reduce infection and assist in temporary wound management. Its role in routine use remains controversial, and further high-quality studies are needed to clarify patient selection and long-term cost-effectiveness.

## Introduction and background

Open fractures represent a significant therapeutic dilemma in skeletal trauma management, with high risks of infection, soft-tissue loss, and long-term disability [1,2]. Globally, musculoskeletal injuries represent a major public health burden, with fractures affecting over 130 million people annually. Open fractures are especially problematic due to their high risk of infection, non-union, and long-term disability, and this burden is magnified in low- and middle-income countries, where limited trauma systems and specialist access contribute to worse outcomes [3-5]. Immediate surgical intervention, skeletal fixation, and optimal wound care protocols are critical to reducing complications and improving outcomes [6]. Conventional dressings, while widely used, may be limited in their ability to control contamination and promote granulation tissue formation.

Negative pressure wound therapy (NPWT), initially characterised by Morykwas and Argenta during the 1990s, has since gained widespread adoption in trauma and reconstructive surgery as a means of enhancing wound healing through tissue approximation forces, cellular stimulation, enhanced circulation, and decreased microbial colonisation [7,8]. In the context of open fractures, particularly high-energy injuries, NPWT has been proposed as an adjunct that may reduce deep surgical-site infection, facilitate earlier soft-tissue coverage, and improve limb salvage rates [9-11]. However, evidence remains mixed, with some randomised controlled trials reporting significant benefits while larger multicentre studies, such as the WOLLF trial, did not demonstrate improvements in functional outcomes [12].

This review aims to evaluate the current evidence regarding the role of NPWT in the management of open fractures, focusing on its impact on infection rates, healing outcomes, and cost-effectiveness. Recent evidence (2024-2025) has also explored portable NPWT devices, novel foam interfaces, and integration of patient-reported outcomes, underscoring its evolving role in contemporary trauma care. This review provides an updated synthesis of contemporary data to contextualise NPWT’s evolving role in trauma care.

## Review

Mechanism and rationale

This modality functions by optimising the wound microenvironment through macro-scale tissue approximation and reparative tissue development. Sustained or cyclical pressure application generates macro-scale tissue approximation alongside micro-scale cellular activation, promoting neovascularisation and proliferative cellular responses [8]. NPWT has also been shown to improve local perfusion, reduce oedema, and decrease bacterial colonisation [7]. These mechanisms collectively enhance granulation tissue formation and may help lower infection risk in contaminated open fractures. However, despite these mechanistic advantages, concerns have been raised regarding its overuse, variable clinical effectiveness, and uncertain cost-effectiveness, highlighting the need for careful patient selection and context-specific application.

Methods and search strategy

A comprehensive bibliographic investigation was performed using PubMed, Google Scholar, and Cochrane Library to identify studies published between January 1997 and August 2025. The following keywords and Boolean operators were applied in different combinations: “negative pressure wound therapy”, “vacuum-assisted closure”, “NPWT”, “open fracture”, “orthopaedic trauma”, and “wound infection”.

Priority was given to randomised controlled trials, prospective cohort studies, and systematic reviews/meta-analyses that evaluated the role of NPWT in the management of open fractures. Foundational mechanistic studies and key narrative reviews were included to provide background context. Reference lists of relevant systematic reviews and meta-analyses were also screened to ensure additional important studies were not missed. Animal studies, case reports without sufficient clinical details, and articles not published in English were excluded.

This review was conducted as a focused narrative review rather than a full systematic review and therefore did not follow the Preferred Reporting Items for Systematic Reviews and Meta-Analyses (PRISMA) or other systematic review reporting frameworks. The absence of formal protocol registration and structured bias assessment is acknowledged as a methodological limitation. Grey literature, unpublished studies, and formal risk of bias assessment tools (e.g., Cochrane Risk of Bias (RoB)) were not applied. Therefore, publication bias and study heterogeneity may not be fully accounted for. Although no formal risk-of-bias tool (e.g., Cochrane RoB and GRADE (Grading of Recommendations, Assessment, Development, and Evaluations)) was applied, studies were informally appraised according to design hierarchy and sample size as a modified evidence quality assessment. The key clinical studies evaluating NPWT in open fractures are summarised in Table 1.

**Table 1:** Summary of evidence on negative pressure wound therapy (NPWT) in open fracture management. NPWT: negative pressure wound therapy; SSI: surgical site infection; QoL: quality of life.

Study (year)	Methodology	Patient cohort	Intervention details	Primary endpoints	Clinical significance
Stannard et al. (2009) [11]	Randomised controlled trial (single-centre)	63 severe open fractures	NPWT vs. gauze dressing	Deep infection rate (infection rates demonstrated a substantial reduction of 5.4% versus 28%), reoperation rate, and healing time	NPWT reduced infection and reoperations; no effect on healing time
Blum et al. (2012) [9]	Prospective cohort study	169 open tibial fractures (Gustilo II–III)	NPWT vs. standard dressing	Deep infection rate (8.4% vs. 20.6%), time to union	NPWT reduced infection; union timeframes showed comparable patterns
Costa et al. (WOLLF trial) (2018) [12]	Multicentre randomised controlled trial	460 severe open lower-limb fractures	NPWT vs. standard dressing	Disability at 12 months, infection, quality of life, and cost	No improvement in disability, infection, or QoL; higher cost
Grant-Freemantle et al. (2020) [10]	Systematic review & meta-analysis	7 studies, 1,095 patients	NPWT vs. standard dressing	Infection, flap failure, union, and reoperation rates	NPWT reduced infection and flap failure; no benefit for union or reoperation
Liu et al. (2024) [13]	Meta-analysis	18 studies (trauma subgroup)	NPWT vs. conventional dressing	Deep SSI (odds ratio of 0.64, 95% CI: 0.52-0.80, P = 0.0001), non-union	NPWT reduced deep SSI; no effect on non-union
Alves et al. (2024) [14]	Systematic review & meta-analysis	Open fractures (especially Gustilo III)	NPWT vs. standard dressing	Infection, hospital stay	NPWT is suggested to reduce infection and hospital stay; evidence is limited in high-grade injuries

Discussion

Infection Rate

Deep surgical-site infection remains the most critical complication following open fractures. Early randomised studies, such as Stannard et al. [11], reported a significantly lower infection rate with NPWT compared with standard gauze dressings. Blum et al. [9] also demonstrated reduced infection rates in a prospective cohort of open tibial fractures. Several meta-analyses reinforce this trend, with Grant-Freemantle et al. [10] and Liu et al. [13] all reporting lower odds of deep infection in patients managed with NPWT. However, the largest multicentre trial, the WOLLF study [15], found no significant difference in infection rates or functional outcomes at one year. More recently, an international Cochrane review [5] and a network meta-analysis by Kim et al. [16] similarly concluded that while NPWT may lower infection in high-risk wounds, the certainty of the evidence remains low to moderate. Overall, the balance of evidence suggests a potential infection-reducing effect of NPWT, although results are inconsistent and may depend on fracture severity and timing of coverage. The heterogeneity across studies may reflect differences in fracture severity (Gustilo grade II vs. III), timing of soft-tissue coverage, variations in NPWT protocol (pressure settings, dressing type, duration), and outcome definitions (superficial vs. deep surgical-site infections). Observational cohorts may also overestimate benefit due to selection bias. Recent pooled analyses (2024-2025) using updated statistical frameworks continue to reinforce a modest infection-reducing effect, although certainty remains moderate.

Healing Time

Evidence regarding fracture healing time under NPWT is limited and inconclusive. Blum et al. [9] reported no significant difference in time to union between NPWT and conventional dressings. Likewise, systematic reviews have not demonstrated consistent improvements in union rates or healing times [13]. A Cochrane overview emphasised that most available trials were not adequately powered to assess fracture union, limiting firm conclusions [17]. This suggests that while NPWT may optimise the wound environment, its direct impact on fracture biology and union is minimal.

Orthoplastic Principles and the “Fix-and-Flap” Paradigm

The orthoplastic model (combined orthopaedic and plastic surgery) underpins modern care. Historical series introduced the “fix and flap” approach, advocating aggressive debridement, stable fixation, and early flap coverage for severe open tibial fractures, with lower infection and amputation rates when performed promptly. NPWT fits into this pathway as a temporary adjunct, not a substitute for early coverage [18].

Flap/Graft Requirement

Soft-tissue management is central to the outcome of severe open fractures. NPWT has been proposed as a useful bridge to definitive flap or graft coverage by maintaining a cleaner wound bed and promoting granulation. Grant-Freemantle et al. [10] reported reduced flap failure rates in the NPWT group, and other reviews, including Alves et al. [14], support this benefit. Kim et al. [16] and a BMJ evidence synthesis by Cook et al. [19] also suggested that NPWT may reduce the need for secondary reconstructive procedures. However, current guidelines still stress that NPWT should not delay early definitive soft-tissue coverage, which remains the gold standard in open fracture management.

From a clinical perspective, NPWT may be most appropriate in severe Gustilo III injuries with substantial soft-tissue loss, where it can serve as a temporary bridge to coverage. Routine use in lower-grade open fractures is less well supported. Timing is also critical: NPWT should not delay early flap coverage, which remains the gold standard.

**Figure 1:**
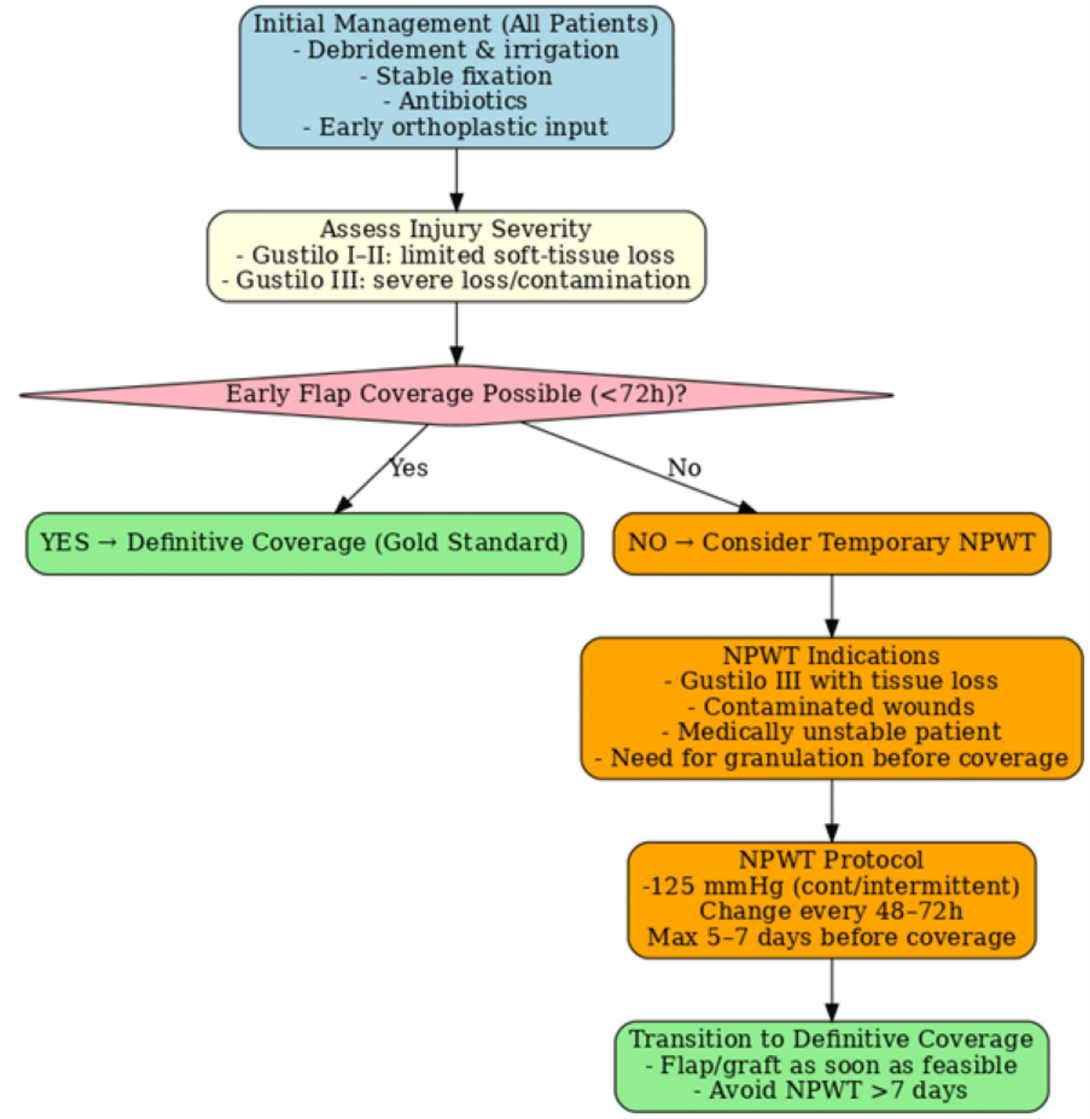
Clinical algorithm for the use of negative pressure wound therapy in open fracture management. Negative pressure wound therapy (NPWT) should be considered as a temporary adjunct in severe Gustilo III injuries where early definitive flap or graft coverage is not feasible within 72 hours. It is not a substitute for timely debridement, stable fixation, and early coverage, which remain the gold standard. The algorithm summarises patient selection, timing, and protocol considerations for practical clinical application. Image credit: Created by the authors. No external sources were used; no permission required.

Figure 1 presents a pragmatic algorithm demonstrating that while NPWT can stabilise the wound environment in high-grade injuries, its role is strictly transitional, with timely debridement and soft-tissue reconstruction remaining the cornerstones of care.

Patient Selection: When NPWT Helps (and When It Does Not)

NPWT is most defensible in Gustilo-Anderson grade III injuries with substantial soft-tissue loss, contamination, or oedema where definitive coverage cannot be achieved at the index debridement [9,20]. In such cases, NPWT can maintain a clean wound bed and reduce dressing burden as a bridge to early flap or graft coverage [21], but it should be regarded only as a temporary adjunct and must not delay definitive soft-tissue reconstruction. Godina first demonstrated that flap coverage performed within 72 hours reduced infection and flap failure [22], and Gopal et al. subsequently introduced the “fix-and-flap” approach, advocating early combined skeletal fixation and flap coverage [18]. More recent studies and systematic reviews confirm that coverage within 72 hours achieves superior outcomes, including lower infection rates and fewer complications [23-25]. Relative contraindications include untreated devitalised tissue, inadequately debrided necrosis, uncontrolled ischaemia prior to revascularisation, active bleeding, and inability to achieve an airtight seal. In lower-grade open fractures with limited soft-tissue compromise, routine NPWT is less clearly justified [26].

Reoperation Rate

The impact of NPWT on reoperation rates is variable across studies. Stannard et al. [11] reported fewer reoperations with NPWT, whereas the WOLLF trial by Costa et al. [15] demonstrated no difference compared with standard dressings. Systematic reviews and guideline updates [5] have not shown consistent reductions in reoperation rates. The disparity may reflect differences in surgical protocols, definitions of reoperation, and timing of soft-tissue coverage across studies.

Cost-Effectiveness

The economic implications of NPWT remain controversial. Health economic analysis alongside the WOLLF trial concluded that NPWT was not economically efficient, as it was associated with higher healthcare costs without improved functional outcomes [27]. A subsequent BMJ analysis by Cook et al. [19] suggested that any potential savings are dependent on reduced infection rates in severe injuries, which has not been consistently demonstrated in large randomised controlled trials. Atwan et al. [28] similarly highlighted the uncertainty surrounding NPWT’s cost-effectiveness, particularly in resource-limited healthcare systems. While reduced infection and reoperation rates could theoretically lower costs, these benefits have not been robustly confirmed in high-quality multicentre studies.

Cost-effectiveness is likely context-dependent. In high-income settings, increased upfront device costs may be offset if deep infection reduction is achieved, whereas in resource-limited systems, affordability and device availability remain major barriers. Formal health economic modelling beyond single-trial analyses is needed. Preliminary health-economic models suggest potential cost neutrality if infection reductions exceed a 10% absolute risk threshold; however, this requires validation across healthcare systems with differing resource utilisation patterns.

Implementation also varies globally, with limited access to NPWT devices in low-resource settings, raising equity concerns. Sustainability considerations, such as device reusability and waste reduction, are increasingly relevant. Emerging technologies, including AI-assisted wound assessment and predictive analytics, may further refine patient selection and protocol standardisation in the near future.

Emerging Technologies and Portable NPWT Devices

Recent innovations have introduced portable and single-use NPWT (suNPWT) systems, designed to improve patient mobility and reduce resource requirements. These devices have been shown to provide comparable wound healing outcomes in surgical and trauma settings, with added benefits such as reduced dressing burden and improved patient comfort [29]. Early studies incorporating patient-reported outcome measures (PROMs) suggest that patient satisfaction and ease of use may be enhanced, although robust data in the context of severe open fractures are still lacking [30]. Further research is needed to clarify whether portable NPWT devices can deliver similar infection reduction and cost-effectiveness benefits as conventional systems in high-grade injuries.

Limitations

This review was conducted as a narrative review rather than a formal systematic review. Only three databases were searched, and no formal risk of bias or quality grading system was applied. Publication bias and heterogeneity may therefore limit the strength of conclusions. In addition, the exclusion of grey literature and unpublished studies may have introduced further bias. The absence of formal grading systems such as GRADE also limits the ability to weigh evidence certainty. Despite these limitations, the review highlights key clinical trends and areas requiring further study.

## Conclusions

Sub-atmospheric pressure treatment has become an important adjunct in the management of complex skeletal injuries, with a strong mechanistic rationale and early clinical studies suggesting reduced deep infection rates and improved wound healing. Randomised controlled trials demonstrated significant benefits, while subsequent larger multicentre investigations failed to show improvements in long-term functional outcomes. Systematic reviews and meta-analyses generally support a reduction in surgical-site infection and flap failure, although results remain heterogeneous and the certainty of evidence is moderate at best. Overall, NPWT appears most beneficial in severe open fractures with extensive soft-tissue loss, where it may serve as a temporary bridge to definitive coverage and limb reconstruction. Its universal application remains controversial, particularly given concerns about resource utilisation optimisation and inconsistent trial findings. Future high-quality randomised studies are needed to better define patient subgroups that derive the greatest benefit, optimise treatment protocols, and establish the long-term economic impact of NPWT in orthopaedic trauma.

Clinicians should consider NPWT primarily in high-energy open fractures with significant soft-tissue compromise. Early debridement, stable fixation, and timely flap coverage remain the pillars of management, with NPWT serving as an adjunct rather than a replacement. Future trials should stratify by fracture severity and protocol standardisation to better define optimal use.
